# Predictors of Undergoing Colonoscopy, Does Time Horizon Matter?

**DOI:** 10.1007/s10935-020-00581-5

**Published:** 2020-01-24

**Authors:** Agnieszka Olchowska-Kotala, Beata Bajcar

**Affiliations:** 1 Department of Medical Humanities and Social Science, Wroclaw Medical University, ul. Mikulicza-Radeckiego 7, 50-367 Wrocław, Poland; 2 Psychology and Ergonomics Group, Faculty of Computer Science and Management, Wrocław University of Science and Technology, Wrocław, Poland

**Keywords:** Risk communication, Time horizon, Colonoscopy, Risk perception, Health Belief Model, Discomfort

## Abstract

When talking to patients about undergoing diagnostic colonoscopy, their doctors can present the risk of developing colorectal cancer (CRC) in different time horizons. Studies on time horizons suggest that people have different psychological associations for the near and distant future, which potentially influence their judgments and actions. The aim of this study was to examine what factors predict patients’ intentions to undergo diagnostic colonoscopy. We particularly focused on examining the role of the time horizon in which the probability of developing malignant CRC was presented, when taking into account the following factors: the perception of risk (perceived susceptibility to and perceived severity of CRC), expected discomfort related to the procedure, a previous colonoscopy, and subjective numeracy. Using the Health Belief Model, we sought to determine whether the intention to undergo a preventive colonoscopy is affected by the time horizon. We hypothesized that the risk of developing CRC in a proximal time horizon would be more threatening to an individual than a distal one and would consequently increase an individual’s behavioral intention to undergo a colonoscopy. We examined the effects of two different time horizons: the risk of developing a disease in the next few years and total lifetime risk. A total of 144 respondents (77 women and 67 men) aged 50–59 years participated in the study. We found that risk perception and expected discomfort significantly affected participants’ intention to undergo a colonoscopy. No empirical evidence was found to confirm that presenting a person with the risk of developing malignant CRC in the coming years, as compared to their lifetime risk, increases the behavioral intention to undergo a diagnostic colonoscopy.

## Introduction

Randomized trials in people of average risk of developing colorectal cancer (CRC) invited to participate in screening tests have showed a reduction in CRC incidence and mortality (von Karsa et al., 2013). According to these investigators, “CRC screening programmes can only be successful if they ensure that as many people as possible in the target population receive the relevant information to enable them to make an informed decision about attending screening” (p. 44). Patients who may be unaware of being in a high risk group or unfamiliar with available diagnostic screenings may be more willing to undergo diagnostic tests if their provider presents them with information about the risk of developing CRC and describes the course of the whole procedure. Colonoscopy is one of the diagnostic procedures performed to detect CRC. Although effective, patients avoid this test due to embarrassment and discomfort (Nelson & Schwartz, 2004). Patients may decide to undergo screening tests for the following reasons: the presence of the disease in their family (McCaul, Branstetter, Schroeder, & Glasgow, 1996), the costs and benefits of the diagnostic screening (McCaul & Tulloch, 1999; Pignone, Bucholtz, & Harris, 1999), socioeconomic status (Whitaker et al., 2011); and a doctor’s recommendation to undergo the procedure (Peterson et al., 2016).

According to many studies, the perception of risk plays a very important role in adopting preventive behaviors. People who perceive that they face more risk of developing a disease are more likely to take preventive measures (Carman & Kooreman, 2014; McCaul & Tulloch, 1999). The perception of risk is closely related to the Health Belief Model (HBM; Hochbaum, Rosenstock, & Kegels, 1952/2016), which is the most frequently cited model of health-related screening behavior. The HBM consists of four main constructs, i.e., perceived susceptibility (belief that one is likely to contract a disease), perceived severity (belief that the disease would have serious consequences), and perceived benefits of and barriers to taking action (Rosenstock, 1966, 1974). Each of these constructs, individually or in combination, can be used to explain health behavior (Sulat, Prabandari, Sanusi, Hapsari, & Santoso, 2018). The HBM proposes that people are most likely to take preventive action if they have high perceived susceptibility and perceived severity in regards to a health problem and if there are fewer costs than benefits to engaging in it (Rosenstock, 1966). Therefore, according to this model, the health promoting behavior is determined by the manner in which the person assesses the relationship of potential losses to potential profits. The HBM emphasizes the subjectivity of this assessment and therefore the key concept of HBM is perception, not objective risks.

It is well-known that rationality is limited when individuals make decisions. Making a decision whether to undergo a screening test, similarly to any other decision, is not always optimal. Tversky and Kahneman (1974) found that when people make decisions in an uncertain situation, they do not analyze all available information. People base their decisions on data that allow them to easily assess a situation and decide what is best for them. Since people are not good intuitive statisticians when confronted with a difficult decision, they do not make rational calculations but instead rely on a limited number of heuristic principles which reduce the complex tasks of assessing probabilities and predicting values to simpler judgmental operations. These heuristics work well under most circumstances, and this may explain their evolutionary development, but in certain cases they lead to systematic errors or cognitive biases (Kahneman, 2011). In other words, people often do not take into account all relevant information when making decisions, but they make decisions on the basis of information that draws their attention, i.e., more accessible information (Kahneman, 2003). The accessibility of information is determined by many factors such as salience, affective valence, similarity, and novelty. The way in which the problem is presented (e.g., the context in which the information is presented or framed) makes certain information more accessible. Therefore, the way in which the information about the possibility of getting sick is presented may influence risk perceptions.

Risk perception is affected by the ability to understand probability and risk information (i.e., numeracy). Numeracy is an element of literacy that refers to the ability to understand numbers (Peters, Hibbard, Slovic, & Dieckmann, 2007). Numeracy, defined in the broadest sense, is the ability to comprehend, use, and attach meaning to numbers (Nelson, Reyna, Fagerlin, Lipkus, & Peters, 2008). Health-related studies also refer to health numeracy as a degree to which individuals have the capacity to access, process, interpret, communicate, and act on numerical, quantitative, graphical, biostatistical, and probabilistic health information needed to make effective health decisions (Golbeck, Ahlers-Schmidt, Paschal, & Dismuke, 2005). Studies on CRC incidence have indicated that people with low numeracy are less knowledgeable about CRC and less likely to undergo screening tests (Smith et al., 2016; Ciampa, Osborn, Peterson, & Rothman, 2010), although the relationship between numeracy and screening behaviors has not been reported in all studies (Schapira et al., 2011).

Another factor, less often taken into account in studies of preventive behaviors, is the temporal distance until the onset of developing a disease. According to construal-level theory (CLT), the perception of temporal distance from an event affects information processing, and consequently, our choices and decisions (Liberman & Trope, 1998). The CLT proposes that temporal distance (as one of the dimensions of psychological distance) changes people’s responses to future events by changing the way people mentally represent those events. The greater the temporal distance, the more likely events are to be represented in terms of a few abstract features that convey the perceived essence of the events (high-level construals) rather than in terms of more concrete and incidental details of the events (low-level construals; Trope & Liberman, 2003). Therefore, decisions regarding distant future events are likely to be based on their relatively central and abstract features (the overall gist of the situation), whereas decisions regarding near future events are likely to be based on their more incidental and concrete features. When doctors talk to their patients about diagnostic colonoscopy, they often refer to several different statistics such as the risk of developing a disease in 5 or 10 years, or lifetime risk, which is the risk of ever developing a disease. Research on temporal distance suggests that people have different psychological associations for the near and distant future, which potentially drive their judgments and actions in different directions. For instance, if an event will occur in the near future, the subjective risk seems to be higher than if it will occur later (Chandran & Menon, 2004).

Our study investigated which factors determine the intention to undergo diagnostic colonoscopy, and in particular the role of time horizon in which the risk of developing CRC is presented. We examined the influence of the presented time horizon (closer vs more distant future) among such factors as the perceived susceptibility to CRC, perceived severity of CRC, expected discomfort, earlier performance of the procedure, and subjective numeracy. We examined effects of two different time horizons: the risk of developing a disease in the coming years and in the lifetime. On the basis of the CLT (Liberman & Trope, 1998) we assumed that if the risk is construed as more proximal and concrete, it will be more likely to enhance the effectiveness of health information. A proximal time horizon may be more threatening to an individual than a distal one and will consequently increase individual’s behavioral intention to undergo a preventive colonoscopy.

## Methods

### Participants

The survey was conducted in southern Poland with a convenience sample of participants who were recruited from a population of adults attending outpatient clinics by trained research assistants. A total of 144 respondents (77 women and 67 male) aged 50–59 years (*M* = 53.4; *SD* = 2.8) participated in the study. The majority of respondents had not undergone a colonoscopy (78%), and 38% of respondents had a chronic disease. Participants’ educational level was as follows: 16% had basic/vocational education, 26%, completed secondary school and 58% had graduated from college or university. To be eligible for the study, participants had to be: (a) aged 50–59 years, (b) literate (individuals with signal difficulties in reading or interpreting questions were excluded), and (c) lacking any medical condition that would affect their ability to participate. All participants were informed about the purpose of the study and provided informed consent.

### Procedure and Measures

We gave all study participants a text describing the frequency of developing CRC (American Cancer Society, 2015) and the possibility of undergoing a colonoscopy to detect asymptomatic changes (a copy of the scenario is available from the corresponding author upon request). The total sample was randomly divided into two conditions. Respondents in the first condition were presented with information on the risk of developing malignant CRC within their age group, i.e. 50–59 years (1 in 193 women and 1 in 148 men). Respondents in the second condition were presented with information on their lifetime risk of developing malignant CRC (1 in 22 women and 1 in 21 men). Based on this information, participants were asked to specify what they perceived as the real likelihood that they might undergo a colonoscopy within the following year. Answers were given on a 5-point scale (1—definitely not, 2—probably not, 3—possibly, 4—very probably, 5—definitely). Next, participants assessed these two items: perceived susceptibility to developing CRC (the probability of an event) on a 10-point scale (from 1—very small to 10—very big), and the perceived severity of CRC (from 1—not a serious disease to 10—a very serious disease). We also asked about participants’ perceived barriers to obtaining a colonoscopy, i.e., the expected discomfort of undergoing the procedure, (from 1—very small to 10—very big). In addition, participants fulfilled the Subjective Numeracy Scale (Fagerlin et al., 2007) which measures the level of perceived ability to perform various mathematical tasks and preferences for using numerical versus textual information. The scale included 8 items rated on a 6-point scale (from 1—not good at all to 6—extremely good).

The survey contained questions about demographic variables (e.g., age, sex, and educational level). In addition, participants completed information about previous colonoscopies, and suffering from chronic diseases.

### Analysis

We performed a hierarchical regression analysis using SPSS statistical software, version 24.0, to establish predictors of intention to undergo colonoscopy. At Step 1, socio-demographic variables and objective characteristics of respondents were added to the hierarchical regressions to control these factors. At Step 2, other independent variables were added. The hierarchical regression was conducted using factors in the following stages to predict the decision: (a) Step 1: sex, education, chronic diseases, and previous colonoscopy; and (b) Step 2: time horizon, perceived severity of CRC, perceived susceptibility to CRC, expected discomfort, and subjective numeracy. The criterion for significance was set at *p* < 0.05.

## Results

Descriptive statistics and correlations among the analyzed variables are presented in the Table 1. At Step 1, the hierarchical regression revealed that experience with previous colonoscopies significantly contributed to the regression model, *F*(4, 139) = 3.03, *p *< .05, and accounted for 8% of the variance in intention. Sex, education and chronic diseases did not influence the intention to undergo colonoscopy. Step 2 indicated that perceived severity of CRC, perceived susceptibility to CRC, and expected colonoscopy discomfort were predictors of intention to undergo this procedure. Adding the variables to the regression model explained an additional 27% of the variance in intention to undergo colonoscopy, and this change in *R*^2^ was significant, *F*(9, 134) = 7.85, *p* < .001. The time horizon and subjective numeracy did not influence the significant percentage of variance in intention to undergo colonoscopy. When all independent variables were included in Step 2 of the regression model, previous colonoscopy experience was not a significant predictor of intention to undergo colonoscopy. Together, the three independent variables accounted for 35% of the variance in intention to undergo colonoscopy. Table 2 presents the regression coefficients for all predictors. As Table 2 shows, intention to undergo colonoscopy was associated with the higher perceived susceptibility to CRC, perceived severity of CRC, and the lower expected discomfort of the procedure.

**Table 1:** Descriptive statistics of the measured variables

	Range	*M*	*SD*	1	2	3	4
1. Expected colonoscopy discomfort	1–10	5.56	2.92	–			
2. Perceived severity of CRC	1–10	9.17	1.29	− .06	–		
3. Perceived susceptibility to CRC	1–10	5.88	2.69	− .07	.18^*^	–	
4. Subjective numeracy	20–48	37.40	6.56	− .17^*^	.05	− .11	–
5. Intention of performing colonoscopy	1–5	3.83	1.15	− .38^***^	.24^**^	.41^***^	.13

*N *=144. *p <.05; **p <.01; ***p <.001

*CRC* colorectal cancer

**Table 2:** Results of hierarchical regression analysis for variables predicting intention to undergo colonoscopy

	Step 1		Step 2	
	β	*p*	β	*p*
Step 1				
Sex	− .05	.53	.07	.39
Education	.15	.08	.06	.47
Chronic disease	− .09	.27	− .12	.09
Previous colonoscopy	.20	.02	.01	.85
Step 2				
Time horizon			− .05	.49
Perceived severity of CRC			.18	.02
Perceived susceptibility to CRC			.38	.001
Expected colonoscopy discomfort			− .29	.001
Subjective numeracy			.08	.29
*R*^2^		.08*		.35***
Δ*R*^2^				.27***

*N *=144. *p <.05; ***p <.001

*CRC* colorectal cancer

## Discussion

We sought to determine predictors of study participants’ intention to undergo a diagnostic colonoscopy, particularly the role of a specific time horizon until the onset of developing CRC. Relevant factors influencing the intention to undergo colonoscopy were as follows: perceived susceptibility to CRC, i.e., the subjective probability of developing CRC; perceived severity of CRC; and expected discomfort of colonoscopy. We expected to find that presenting the risk of developing CRC in the coming years increases behavioral intentions to undergo colonoscopy more than presenting lifetime risk, but this hypothesis was not confirmed. The difference in the level of intention to undergo colonoscopy when facing information about the risk of developing CRC in the coming years compared to lifetime risk was not significant. In several previous studies, time horizon also did not affect the intention of behavior (Fair, Murray, Thomas, & Cobain, 2008; Scott & Curbow, 2006). Some research shows that people are indifferent to the time horizon in which risks are presented (Waldron, van der Weijden, Ludt, Gallacher, & Elwyn, 2011). Our research is congruent with these findings. The lack of contribution of this factor may result from the fact that in the message we showed respondents, words such as “malignant” or “colonoscopy” attracted attention and the fact that they referred to either a closer or more distal time perspective was, in this context, less important. Our finding can be interpreted by referring to the accessibility of the information we provided. According to Kahneman (2003), some attributes of a situation are more accessible than others, both in perception and in judgment. He suggests that emotionally arousing stimuli spontaneously attract attention and reduce the accessibility of other stimuli. It seems that the information about the possibility of developing malignant CRC was more salient than the information about the likelihood of developing the disease within a given time horizon. This saliency could be the reason why the intention to undergo a colonoscopy was not affected by presenting the information about the possibility of developing the disease in a short- or long-term time horizon. In many health studies, the way that information has been presented has had no or little significance (Akl et al., 2011), which is due to the fact that patients are actively involved in the matter of their own health (McElroy & Seta, 2003).

Our study indicated that the higher people assess their own susceptibility to CRC and the severity of the disease, the more likely it is that they will decide to undergo a colonoscopy. According to the HBM, an individual’s perceived susceptibility to disease interacts with the perceptions of disease severity and a combination of these two factors is referred to as perceived threat of an illness (Rosenstock, Strecher, & Becker, 1994). The greater the fear, the more likely it is that the person will undertake health-promoting behaviors. Most studies indicate that raising fear and increasing the subjective risk assessment motivates people and encourages them to undertake health-promoting behaviors (Tannenbaum et al., 2015). Some authors, however, emphasize that the increased risk perception associated with the perceived susceptibility to or the severity of the disease can both motivate and inhibit adopting health-promoting behaviors (Schapira et al., 2011). Practitioners and clinicians point out that the relationship between risk perception and the intention to undergo a procedure to detect a disease may be non-linear (Witte & Allen, 2000), and that a very high level of fear may have the opposite effect to the one intended. Due to the fact that cancer worries may pose a barrier to detective behavior (Lerman et al., 1993), it might be best if doctors determined their patients’ subjective perceptions during consultations. We should, however, treat with caution the result that the higher the perceived susceptibility to and severity of CRC, the higher the intention to undergo colonoscopy, knowing that perceiving the risk as too high may lead to developing avoidance strategies.

Another designated predictor of undergoing colonoscopy was the expected discomfort of the procedure. According to the HBM assumptions, it may constitute a barrier to perform this medical procedure. Many studies have shown that discomfort, defined as “potential unpleasant effects from the test,” can determine patients’ decisions to undergo a procedure. In a study of the choice between less and more invasive methods of diagnostic testing (fecal occult blood testing or colonoscopy), in which patients were asked to indicate criteria they use when deciding to undergo a procedure, discomfort was second in the hierarchy. Discomfort was indicated even more frequently than potential adverse events of the test itself (Ling, Moskowitz, Wachs, Pearson, & Schroy, 2001). Previous studies have found that, relative to men, women have particularly negative attitudes towards colonoscopy and are more concerned about the sex of the endoscopist (Farraye et al., 2004). Moreover, women viewed the preparation required for endoscopic procedures as a major barrier to screening (Friedemann‐Sánchez, Griffin, & Partin, 2007). Even though we reported that subjective evaluations turned out to be more important for explaining the intention to undergo colonoscopy than patients’ socio-demographic characteristics, undergoing previous colonoscopy was the most important factor among the objective characteristics we specified. That is, individuals who had previously undergone colonoscopy were more likely to undergo it again than were individuals who had never done it before. In our study, the real past experience of this discomfort did not reduce the intention to undergo it again. In future research, it would be worth determining what information could decrease the expected discomfort. A conversation about sedation and the course of the procedure, or the assurance from doctors that they could choose the sex of the endoscopist as preferred by the patient, could potentially reduce embarrassment and anticipated discomfort.

Our findings confirmed the results of the study of Schapira et al. (2011), which found no association between numeracy and the intention to undergo cancer screening. Previous studies examining the influence of numeracy on undergoing preventive screening tests yielded inconclusive results. Some studies indicated that numeracy is related to medical decisions, including screening tests (Reyna, Nelson, Han, & Dieckmann, 2009), and that low numeracy respondents are less positive towards screening (Smith et al., 2016; Ciampa et al., 2010). However, other studies have suggested somewhat different conclusions (Miron-Shatz, Hanoch, Katz, Doniger, & Ozanne, 2015). The relationship between numeracy and preventive behavior is complex and may be affected by many different factors such as: the degree of mathematical difficulty in the message or and patients’ emotional responses to the subjective perception of risk. In this study, which presented a relatively simple message, we did not confirm that numeracy determines the intention to undergo diagnostic colonoscopy. Further examination of this relationship is needed in order to understand the relevance of numeracy to cancer screening behavior.

### Limitations

There are several limitations to this study. First, our results are based on a convenience sample. Our study included participants in the age group of 50–59 years, and not individuals at high risk of developing CRC, who are most often recommended to undergo a colonoscopy. Secondly, we did not examine real behaviors, but only participants’ intentions, which may or may not be related to the behavior itself. Further research is needed to determine if increased risk perceptions lead not only to increased behavioral intention, but also to actually undergoing the procedure. Moreover, we did not analyze all factors indicated in the literature that may be related to making decisions on performing screening tests, such as a belief that the procedure does not increase the probability of developing a disease or lack of access to medical services. In addition, our sample was relatively small and overrepresented by people with higher education, which also limited the generalizability of study results.

## Conclusions

Despite these limitations, our results suggest that the intention to undergo colonoscopy is determined by subjective risk perception, i.e., the assessment of the susceptibility to and severity of CRC. A high subjective risk of developing CRC and perceiving the disease as serious increases behavioral intention to undergo colonoscopy. This intention, in turn, is reduced by the expected discomfort associated with the procedure. Such results confirm the explanatory power of HBM and its usefulness in the prediction of health-related screening behavior. No empirical evidence was found to confirm that presenting a person with the risk of developing malignant CRC in the coming years, as compared to their lifetime risk, increases their behavioral intention to perform diagnostic colonoscopy. The lack of influence of the presented time horizon on patients’ intention to undergo colonoscopy should be confirmed on a larger sample size, especially with people at the higher risk of developing CRC.

## References

[CR1] Akl EA, Oxman AD, Herrin J, Vist GE, Terrenato I, Sperati F, Costiniuk C, Blank D, Schünemann H (2011). Framing of health information messages. The Cochrane Database of Systematic Reviews.

[CR2] American Cancer Society (2015). Cancer facts and figures.

[CR3] Carman KG, Kooreman P (2014). Probability perceptions and preventive health care. Journal of Risk and Uncertainty.

[CR4] Chandran S, Menon G (2004). When a day means more than a year: Effects of temporal framing on judgments of health risk. Journal of Consumer Research.

[CR5] Ciampa PJ, Osborn CY, Peterson NB, Rothman RL (2010). Patient numeracy, perceptions of provider communication, and colorectal cancer screening utilization. Journal of Health Communication.

[CR6] Fagerlin A, Zikmund-Fisher BJ, Ubel PA, Jankovic A, Derry HA, Smith DM (2007). Measuring numeracy without a math test: Development of the subjective numeracy scale. Medical Decision Making.

[CR7] Fair AK, Murray PG, Thomas A, Cobain MR (2008). Using hypothetical data to assess the effect of numerical format and context on the perception of coronary heart disease risk. American Journal of Health Promotion.

[CR8] Farraye FA, Wong M, Hurwitz S, Puleo E, Emmons K, Wallace MB, Fletcher RH (2004). Barriers to endoscopic colorectal cancer screening: Are women different from men?. American Journal of Gastroenterology.

[CR9] Friedemann-Sánchez G, Griffin JM, Partin MR (2007). Gender differences in colorectal cancer screening barriers and information needs. Health Expectations.

[CR10] Golbeck AL, Ahlers-Schmidt CR, Paschal AM, Dismuke SE (2005). A definition and operational framework for health numeracy. American Journal of Preventive Medicine.

[CR11] Hochbaum, G., Rosenstock, I., & Kegels, S. (1952/2016). *Health belief model.* Washington, DC: U.S. Public Health Service. (Original work published 1952).

[CR12] Kahneman D (2003). Maps of bounded rationality: Psychology for behavioral economics. American Economic Review.

[CR13] Kahneman D (2011). Thinking, fast and slow.

[CR14] Lerman C, Daly M, Sands C, Balshem A, Lustbader E, Heggan T, Goldstein L, James J, Engstrom P (1993). Mammography adherence and psychological distress among women at risk for breast cancer. Journal of the National Cancer Institute.

[CR15] Liberman N, Trope Y (1998). The role of feasibility and desirability considerations in near and distant future decisions: A test of Temporal Construal Theory. Journal of Personality and Social Psychology.

[CR16] Ling BS, Moskowitz MA, Wachs D, Pearson B, Schroy PC (2001). Attitudes toward colorectal cancer screening tests. Journal of General Internal Medicine.

[CR17] McCaul KD, Branstetter AD, Schroeder DM, Glasgow RE (1996). What is the relationship between breast cancer risk and mammography screening? A meta-analytic review. Health Psychology.

[CR18] McCaul KD, Tulloch HE (1999). Cancer screening decisions. Journal of the National Cancer Institute Monographs.

[CR19] McElroy T, Seta JJ (2003). Framing effects: An analytic-holistic perspective. Journal of Experimental Social Psychology.

[CR20] Miron-Shatz T, Hanoch Y, Katz BA, Doniger GM, Ozanne EM (2015). Willingness to test for BRCA1/2 in high risk women: Influenced by risk perception and family experience, rather than by objective or subjective numeracy?. Judgment and Decision Making.

[CR21] Nelson RL, Schwartz A (2004). A survey of individual preference for colorectal cancer screening technique. BMC Cancer.

[CR22] Nelson W, Reyna VF, Fagerlin A, Lipkus I, Peters E (2008). Clinical implications of numeracy: Theory and practice. Annals of Behavioral Medicine.

[CR23] Peters E, Hibbard J, Slovic P, Dieckmann N (2007). Numeracy skill and the communication, comprehension, and use of risk-benefit information. Health Affairs.

[CR24] Peterson EB, Ostroff JS, DuHamel KN, D’Agostino TA, Hernandez M, Canzona MR, Bylund CL (2016). Impact of provider-patient communication on cancer screening adherence: A systematic review. Preventive Medicine.

[CR25] Pignone M, Bucholtz D, Harris R (1999). Patient preferences for colon cancer screening. Journal of General Internal Medicine.

[CR26] Reyna VF, Nelson WL, Han PK, Dieckmann NF (2009). How numeracy influences risk comprehension and medical decision making. Psychological Bulletin.

[CR27] Rosenstock IM (1966). Why people use health services. The Milbank Memorial Fund Quarterly.

[CR28] Rosenstock IM (1974). The health belief model and preventive health behavior. Health Education Behavior.

[CR29] Rosenstock IM, Strecher VJ, Becker MH, DiClemente RJ, Peterson JL (1994). The health belief model and HIV risk behavior change. Preventing AIDS. AIDS prevention and mental health.

[CR30] Schapira MM, Neuner J, Fletcher KE, Gilligan MA, Hayes E, Laud P (2011). The relationship of health numeracy to cancer screening. Journal of Cancer Education.

[CR31] Scott LB, Curbow B (2006). The effect of message frames and CVD risk factors on behavioural outcomes. American Journal of Health Behavior.

[CR32] Smith SG, Kobayashi LC, Wolf MS, Raine R, Wardle J, von Wagner C (2016). The associations between objective numeracy and colorectal cancer screening knowledge, attitudes and defensive processing in a deprived community sample. Journal of Health Psychology.

[CR33] Sulat J, Prabandari Y, Sanusi R, Hapsari E, Santoso B (2018). The validity of health belief model variables in predicting behavioral change. Health Education.

[CR34] Tannenbaum MB, Hepler J, Zimmerman RS, Saul L, Jacobs S, Wilson K, Albarracín D (2015). Appealing to fear: A meta-analysis of fear appeal effectiveness and theories. Psychological Bulletin.

[CR35] Trope Y, Liberman N (2003). Temporal construal. Psychological Review.

[CR36] Tversky A, Kahneman D (1974). Judgment under uncertainty: Heuristics and biases. Science.

[CR37] von Karsa L, Patnick J, Segnan N, Atkin W, Halloran S, Lansdorp-Vogelaar I, European Colorectal Cancer Screening Guidelines Working Group (2013). European guidelines for quality assurance in colorectal cancer screening and diagnosis: Overview and introduction to the full supplement publication. Gastroenterological Endoscopy.

[CR38] Waldron CA, van der Weijden T, Ludt S, Gallacher J, Elwyn G (2011). What are effective strategies to communicate cardiovascular risk information to patients? A systematic review. Patient Education and Counseling.

[CR39] Whitaker KL, Good A, Miles A, Robb K, Wardle J, von Wagner C (2011). Socioeconomic inequalities in colorectal cancer screening uptake: Does time perspective play a role?. Health Psychology.

[CR40] Witte K, Allen M (2000). A meta-analysis of fear appeals: Implications for effective public health campaigns. Health Education & Behavior.

